# Case Report: Dual Inhibition of HDAC and BTK for Diffuse Large B-Cell Lymphoma After Failure to CD19-Targeted CAR-T Therapy

**DOI:** 10.3389/fimmu.2022.894787

**Published:** 2022-06-10

**Authors:** Weiguo Zhu, Shi Tao, Wenchun Miao, Hui Liu, Xianggui Yuan

**Affiliations:** ^1^Department of Hematology, Shaoxing Second Hospital, Shaoxing, China; ^2^Department of Hematology, The Second Affiliated Hospital, College of Medicine, Zhejiang University, Hangzhou, China

**Keywords:** diffuse large B-cell lymphoma, chimeric antigen receptor T cell, chidamide, zanubrutinib, histone deacetylase inhibitor, Bruton’s tyrosine kinase inhibitor

## Abstract

**Background:**

Failure to CD19-targeted chimeric antigen receptor T-cell (CAR-T) therapy for patients with relapsed/refractory (R/R) diffuse large B-cell lymphoma (DLBCL), is an emerging clinical problem. There is no consensus on the treatment for these patients and treatment remains empirical.

**Case Report:**

We reported a case of an elderly R/R DLBCL patient who had *TP53* mutation and relapsed 12 months after initial response to CAR T-cell therapy. The patient did not respond to salvage chemotherapy with the GDP regimen and could not tolerate any aggressive chemotherapy. Thereafter, the patient was given chidamide and zanubrutinib. After two months of treatment, the patient achieved sustained complete remission. At the last follow-up, the patient remains in radiographic CR 22 months after CAR-T infusion and 10 months after the initiation of the combination treatment.

**Conclusion:**

We report the first successful case of dual inhibition of HDAC and BTK for the treatment of R/R DLBCL after failure to CAR-T cell therapy, which opens a new therapeutic possibility for the future.

## Introduction

CD19-targeted chimeric antigen receptor T cell (CAR-T) immunotherapy has been reported with superior response and long-term survival benefits for patients with relapsed or refractory (R/R) diffuse large B-cell lymphoma (DLBCL) (1–3). Despite these encouraging clinical results, more than half of patients either do not respond to or eventually relapse after CAR-T therapy (4, 5). The prognosis of these patients is usually very poor with a median overall survival (OS) of approximately 6 months (6). They represent a new unmet need in the treatment of this disease.

Unfortunately, there are only a few salvage options currently available and there are no standard options for DLBCL patients after CAR-T failure (7). Although novel drugs, such as selinexor, tafasitamab, polatuzumab, checkpoint inhibitors, loncastuximab tesirine, and bi-specific antibodies, are clinically available, there are no data available on the patients with CAR-T failure. It is unclear whether any of these drugs will lead to durable remissions. CAR-T cells targeting an alternative antigen, administration of 1 or more kinds of CAR-T cells targeting different antigens, bivalent CAR-Ts targeting multiple antigens, and allogeneic CAR-T show favorable responses after CD19-targeted CAR-T failure. However, these CAR-Ts are still in clinical trials and only accessible to a small proportion of patients in a few large centers. Allogeneic stem cell transplantation could also be considered in eligible patients. However, the recipient’s fitness and donor availability may impede its applicability immediately after CAR-T relapse. Therefore, there is an urgent demand for novel drugs and drug combinations to treat these patients.

Histone deacetylases (HDACs), a family of enzymes that remove acetyl groups from histones, are critical epigenetic silencers. Histone deacetylase inhibitors (HDACi) function by interfering with HDACs can reverse transcriptional inhibition of tumor suppressor genes. Recently, several kinds of HDACi were evaluated in clinical trials for B-cell non-Hodgkin’s lymphoma (NHL). In a phase 2 study, mocetinostat, an isotype-selective HDACi, achieves a 18.9% overall response rate (ORR) in 41 R/R DLBCL patients, and nearly 1/3 of these patients reach a stable disease state (8). In a retrospective study, chidamide with PEL regimen (prednisone, etoposide, lenalidomide) in 34 unfit patients with R/R DLBCL achieved an ORR of 73.5%, a complete remission (CR) rate of 32.4%, and a 1-year expected progression free survival (PFS) of 43.0% (9). Bruton’s tyrosine kinase (BTK) plays a vital role in the BCR signaling pathway. BTK inhibitors (BTKi) have confirmed therapeutic activity in B-cell malignancies, with modest activity in DLBCL. Ibrutinib, the first-in-class BTK inhibitor, has shown modest activity with an ORR of 37% in patients with R/R non-germinal center B-cell (non-GCG) DLBCL (10). Zanubrutinib, a second-generation BTKi, produced an ORR of 29% and a CR rate of 17% in R/R DLBCL regardless of molecular subtype (11). In preclinical experiments and case reports, dual inhibition of HDAC and BTK showed a possible synergistic effect for DLBCL (12, 13).

We here present a case of an elderly R/R DLBCL patient who relapsed after initial response to CAR T-cell therapy and achieved complete remission after treatment with chidamide and zanubrutinib.

## Case Report

A 70-year-old Chinese man with bilateral axillary lymphadenopathy was diagnosed with diffuse large B-cell lymphoma, not otherwise specified through lymph node biopsy 5 years before this study. Immunohistochemical analysis showed the following results for lymphoma cells: CD3 (-), CD10 (-), CD20 (+), BCL2 (+, 80%), BCL6 (+), C-MYC (+, 40%), CyclinD1 (-), MUM1 (+), CD21 (-) and Ki-67 (+, 80%). EBER by *in situ* hybridization test (ISH) was negative in the lymphoma cells. The fluorescence *in situ* hybridization test (FISH) yielded negative results for MYC, BCL2/IGH and BCL-6 rearrangement. 18F-fluorodeoxyglucose (FDG) positron emission tomography (PET-CT) revealed multiple lymphadenopathies on both sides of the mediastinum with larynx and stomach involvement. Bone marrow biopsy was negative. A diagnosis of DLBCL, non-GCB subtype with MYC/BCL2 double expression was made. The Ann Arbor stage was IV A and the international prognostic index was 3, suggesting a poor prognosis. The patient then received 6 courses of R-CHOP (rituximab, cyclophosphamide, doxorubicin, vincristine, and prednisone) and achieved the first complete remission (CR). The patient declined autologous stem cell transplantation (auto-SCT). However, 9 months later, the patient experienced his first relapse, developing a single lesion on his tongue. The mass biopsy confirmed the relapse. Two courses of dose-adjusted R-EPOCH (etoposide, doxorubicin, cyclophosphamide, vincristine, prednisone, and rituximab) and two courses of R-DHAP (rituximab, dexamethasone, high-dose cytarabine, and cisplatin) only allowed the patient to achieve stable disease (SD). Therefore, involved field radiotherapy was given and the patient reached his second CR. However, 7 months later, the patient relapsed for the second time, showing bilateral cervical lymphadenopathy confirmed by biopsy. Four courses of L-GemOx (lenalidomide, gemcitabine and oxaliplatin) were given and the patient reached CR as confirmed by PET-CT evaluation. Then, lenalidomide was given as maintenance treatment. However, another 7 months later, the patient relapsed for the third time with a new lung mass confirmed by biopsy as DLBCL, which was CD19 positive.

**Figure 1:**
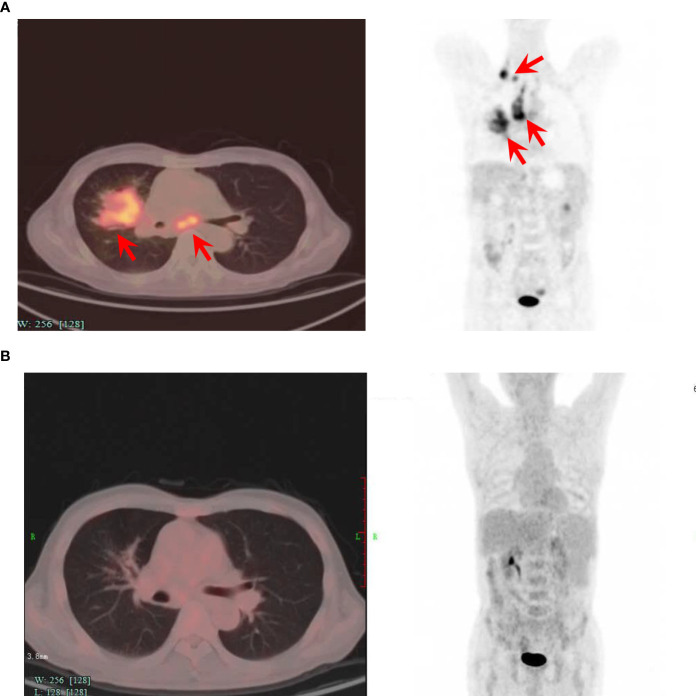
Positron-emission tomography-computed tomography before and 3 months after CAR-T cell infusion. **(A)** PET-CT image of the patient before the CAR-T cell infusion, showing lymphoma invasion. **(B)** PET-CT image of the patient 3 months after the CAR-T cell infusion, showing no lesions and complete remission. The axial image (left) and coronal image (right) are shown. Red arrows indicate lymphoma lesions.

The patient was then referred to our center and recruited in a clinical trial of CD19-targeted CAR-T therapy with costimulatory 4-1BB endodomain (ClinicalTrials.gov ID: NCT04833504). Pretreatment PET-CT showed a large lung mass with multiple lymph node involvements (**Figure 1A**). The patient underwent FC regimen lymphodepletion chemotherapy (fludarabine 30 mg/m^2^ day -5 to -3, cyclophosphamide 500 mg/m^2^ day -5 to -3) before CAR-T infusion. The patient was infused with a total of 1.1×10^8^ CAR-T cells (2×10^6^ cells per kilogram) on day 0. After CAR-T infusion, the patient suffered from grade 1 cytokine release syndrome (CRS) without immune effector cell-associated neurotoxicity syndrome (ICANS) according to the American Society for transplantation and cellular therapy (ASTCT) guidelines. PET-CT examination confirmed complete metabolic remission 3 months after CAR-T cell therapy (**Figure 1B****)**.

**Figure 2:**
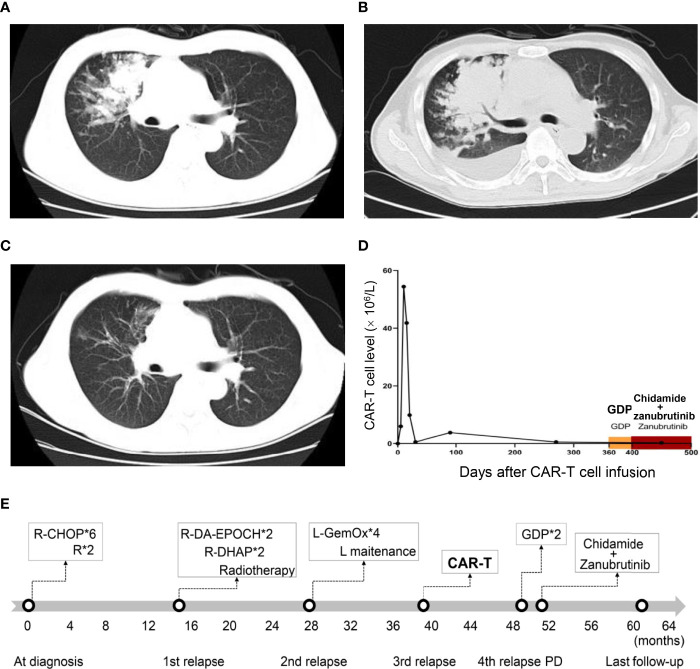
Computed tomography and CAR-T cell monitoring after CAR-T infusion. **(A)** Axial CT image at relapse after CAR-T therapy. **(B)** Axial CT image before the combination treatment of chidamide and zanubrutinib, showing disease progression. **(C)** Axial CT image 2 months after the combination treatment of chidamide and zanubrutinib, showing disease remission. **(D)** CAR-T cell monitoring after CAR-T cell infusion. **(E)**Timeline of diagnosis and treatment. “*” means “times” (or multiply).

Unfortunately, 12 months after CAR-T infusion, the patient relapsed for the fourth time with lung mass (**Figure 2A**), which was confirmed by biopsy. Notably, immunohistochemical analysis showed that CD19 was still positive. Circulating tumor DNA (ctDNA) of DLBCL (a 49 cancer-related gene panel, **Supplementary File 1**) performed with next-generation sequencing (Illumina, Wuhan Sitaide Medical Laboratory) detected the mutations in *TP53* (c.524G>A, p.R175H, 47.60%) and *NOTCH1* (c.3539G>A, p.C1180Y, 35.02%), without mutations in MYD88, CD79b, EP300 or CREBBP. During the salvage chemotherapy with two courses of GDP regimen, the patient suffered from grade IV bone marrow suppression and infective fever. However, the patient failed to achieve any remission and lung lesion progressed with increasing pleural effusion (**Figure 2B**). His chest distress and shortness of breath worsened, and he could not tolerate any further aggressive chemotherapy. Thereafter, He was given oral chidamide 30 mg twice a week and oral zanubrutinib (a novel BTK inhibitor) 160mg twice daily as salvage therapy. Surprisingly, his symptoms were relieved after 2 weeks of this treatment, and a CT scan 2 months later showed significant remission of lung lesions (**Figure 2C**). This regimen was well tolerated with only grade 1 leukopenia. Peripheral blood CAR-T cells of the entire course management were monitored of this patient (**Figure 2D****)**. There were no CAR-T cells by flow cytometry and CAR copies by ddPCR detected at relapse after CAR-T therapy and there was no CAR-T cell expansion at the remission of this regimen. At the last follow-up, the patient remains in radiographic CR at 22 months after CAR-T infusion and 10 months after the initiation of chidamide and zanubrutinib. The timeline of diagnosis and treatment was shown in **Figure 2E**.

## Discussion

Loss of tumor antigens and CAR-T cell exhaustion contributed to the adaptive resistance to CAR-T immunotherapy (14). Following CAR-T, CD19 loss occurs in approximately 30% of cases at time of progression (6), however, its mechanism is unclear. CAR-T cell exhaustion could partially attribute to epigenetic dysregulation, such as histone acetylation modification. The pan-HDAC inhibitor (LAQ824) can decrease T regulatory cell populations and enhance adoptive T cell therapy survival, which significantly improves the antitumor immune activity in recipient mice (15). The class I HDACi (entinostat) enhances the proliferative and functional capacity of CD8+ T cells and sensitizes tumor cells to T-cell recognition, which improves the antitumor effect (16). The HDACi (LBH589 and SAHA) partially reversed the resistance of lymphoma cells to CD19-targeted CAR-T cells (17). BTK inhibitors inhibit AKT signaling, reduce cell terminal differentiation, increase the proportion of memory CAR-T cells, and consequently, increase the efficiency of CD19-targeted CAR-T cell therapy (18). For our case, CD19 was persistently positive, so CAR-T cell exhaustion might be the primary cause. Since we did not observe a significant expansion of CAR-T cells, the antitumor effect of dual inhibition of HDAC and BTK therapy might not rely on promoting T-cell clonal expansion or enhancing the efficacy of CAR-T cells.

Chidamide, which selectively inhibits the activity of HDAC1, HDAC2, HDAC3, and HDAC10, has been approved by the Chinese FDA to treat R/R peripheral T-cell lymphomas. It is the only orally active HDACi available in the Chinese market (19). Chidamide has been used in combined treatments for newly diagnosed and R/R DLBCL in many clinical trials. Zanubrutinib, a potent and selective BTK inhibitor, has been approved for the treatment of R/R chronic lymphocytic leukemia and mantle cell lymphoma with a trend toward better responses and less toxicity, particularly cardiovascular toxicities (20, 21). We gave this patient chidamide and zanubrutinib, mainly based on the accessibility and lower off-target toxicities. The regimen was well tolerated and the patient again achieved remission. Similarly, it was reported that combination treatment with chidamide and ibrutinib produced complete remission in a patient with primary refractory DLBCL (12). Nevertheless, the mechanism underlying the synergistic mechanism remains unclear.

There are connections between the BTK signaling pathway and HDACs. HDACi restores the expression of BTK-targeting miRNA, resulting in decreased signaling of the BTK pathway and ultimately apoptosis (22). HDAC6 inhibition acetylates and abrogates the effects of MYD88, leading to cell cycle arrest and cell apoptosis (23). Tonic HDAC6 inhibition upregulates the IRE1 pathway of the unfolded protein response, leading to up-regulation of the BCR pathway (24). In preclinical models, HDACi transcriptionally downregulates *MyD88* transcription, moreover, combining ibrutinib with panobinostat (a HDACi) resulted in synergistic inhibition of NF-κB activity and caused regression of DLBCL xenografts (13). In this way, dual inhibition of HDAC and BTK could theoretically have a synergistic anti-lymphoma effect. Many DLBCL patients harbor *NOTCH1* mutations, termed N1 genetic subtypes (25). *NOTCH1* promotes the activation of the NF-κB signaling pathway and PI3K-AKT-mTOR, which plays a vital role in promoting cell growth and accelerating cell apoptosis in lymphoma cells. Combination treatment with ibrutinib and enzastaurin significantly reduces the expression of *NOTCH1*, which dramatically inhibits DLBCL cell proliferation (26). *TP53* mutations, occurring in approximately 20-30% of DLBCL patients, are associated with poor prognosis and therapy refractoriness. There is no standard therapy for the treatment of DLBCL with *TP53* mutation. HDAC1 and HDAC2 can restore the expression of mutant *TP53*, and HDACi can reduce the expression of mutant *TP53* (27). Chidamide has a therapeutic effect on DLBCL by upregulating the surface expression of the CD20 antigen and inhibiting the expression of mutant *TP53* (28). Our patient did not have mutations in *CREBBP*, *EP300*, or *MYD88(L265P)*, which have been reported as targets for HDCA inhibitors or BTK inhibitors, respectively. However, our patient had *TP53* and *NOTCH1* mutations, which we considered may be the targets of dual inhibition of HDAC and BTK. The precise underlying mechanism of dual inhibition of HDAC and BTK is likely to be a promising direction for further research.

Our case has several limitations. First, the potential synergistic mechanism of the dual inhibitors was not well clarified. Second, the 10 months of follow-up period is relatively short, so whether dual inhibitor treatment will lead to long-term remission is unclear. However, we reported the first successful case of dual inhibition of HDAC and BTK for the treatment of R/R DLBCL after failure to CAR-T cell therapy, which opens a new therapeutic possibility for the future.

In conclusion, dual inhibition of HDAC and BTK may serve as a salvage therapeutic strategy for DLBCL patients relapsed or refractory to CAR-T therapy, which deserves further investigation in a large population.

## Data Availability Statement

The original contributions presented in the study are included in the article/**Supplementary Material**. Further inquiries can be directed to the corresponding authors.

## Ethics Statement

The studies involving human participants were reviewed and approved by the Human Ethics Committee of the Second Affiliated Hospital, School of Medicine, Zhejiang University, China (SAHZ2022-0251). The patients/participants provided their written informed consent to participate in this study.

## Author Contributions

Conceptualization: XY and HL; Data curation: WZ and ST; Formal analysis: WM; Funding acquisition: HL; Resources: XY and HL; Supervision: XY and HL; Validation: WZ and ST; Writing – original draft: WZ; Writing – review and editing: XY and HL. All authors contributed to the article and approved the submitted version.

## Funding

This work was supported by the Translational Research Grant of National Clinical Research Center for Hematologic Diseases (NCRCH) (No. 2020ZKZC01), Zhejiang Provincial Natural Science Foundation (No.LY18H160008) and the Lymphoma Research Fund of China Anti-Cancer Association.

## Conflict of Interest

The authors declare that the research was conducted in the absence of any commercial or financial relationships that could be construed as a potential conflict of interest.

## Publisher’s Note

All claims expressed in this article are solely those of the authors and do not necessarily represent those of their affiliated organizations, or those of the publisher, the editors and the reviewers. Any product that may be evaluated in this article, or claim that may be made by its manufacturer, is not guaranteed or endorsed by the publisher.

## Supplementary Material

Supplementary Table 1 A 49 cancer-related gene panel of circulating free DNA of DLBCL.

## Abbreviations

Auto-SCT, Autologous stem cell transplantation; ASTCT, American Society for transplantation and cellular therapy; BTK, Bruton’s tyrosine kinase; BTKi, Bruton’s tyrosine kinase inhibitors; CAR-T, Chimeric antigen receptor T cell; CRS, Cytokine release syndrome; CR, complete remission; HDACs, Histone deacetylases; HDACi, Histone deacetylase inhibitors; ICANS, Immune effector cell-associated neurotoxicity syndrome; NHL, Non-Hodgkin’s lymphoma; Non-GCG, non-germinal center B-cell; ORR, Overall response rate; OS, Overall survival; PET-CT, positron emission tomography-computed tomography; PFS, Progression free survival; R/R DLBCL, Relapsed or refractory diffuse large B-cell lymphoma; SD, Stable disease.
