# Supplementary material for: Case Report: Dual Inhibition of HDAC and BTK for Diffuse Large B-Cell Lymphoma After Failure to CD19-Targeted CAR-T Therapy
Source: Front Immunol. 2022 Jun 10;13:894787. doi: 10.3389/fimmu.2022.894787 (PMC9226330; doi:10.3389/fimmu.2022.894787)
Supplement: Supplementary Table 1 — A 49 cancer-related gene panel of circulating free DNA of DLBCL. [file Table_1.docx]

1.*ARID1A*

2.*CD58*

3.*IKBKB*

4.*NOTCH2*

5.*SPEN*

6.*ATM*

7.*CD79B*

8.*ITPKB*

9.*NRAS*

10.*STAT6*

11.*B2M*

12.*CHD2*

13.*KLHL6*

14.*PCLO*

15.*TBL1XR1*

16.*BCL2*

17.*CREBBP*

18.*KMT2D*

19.*PIK3CA*

20.*TCF3*

21.*BCOR*

22.*DDX3X*

23.*KRAS*

24.*PIM1*

25.*TGM7*

26.*BIRC3*

27.*EGR2*

28.*LRP1B*

29.*POT1*

30.*TNFAIP3*

31.*BRAF*

32.*EP300*

33.*MAP2K1*

34.*PRDM1*

35.*TNFRSF14*

36.*CARD11*

37.*EZH2*

38.*MED12*

39.*RET*

40.*TP53*

41.*CCND1*

42.*FBXW7*

43.*MEF2B*

44.*RIPK1*

45.*WHSC1*

46.*CCND2*

47.*GNA13*

48.*MYC*

49.*SAMHD1*

50.*XPO1*

51.*CCND3*

52.*HIST1H1E*

53.*MYD88*

54.*SF3B1*

55.*ZMYM3*

56.*CD36*

57.*ID3*

58.*NOTCH1*

59.*SIN3A*
